# Comprehensive analysis of immune-related prognostic genes in the tumour microenvironment of hepatocellular carcinoma

**DOI:** 10.1186/s12885-021-08052-8

**Published:** 2021-03-31

**Authors:** Weike Gao, Luan Li, Xinyin Han, Siyao Liu, Chengzhen Li, Guanying Yu, Lei Zhang, Dongsheng Zhang, Caiyun Liu, Erhong Meng, Shuai Hong, Dongliang Wang, Peiming Guo, Guangjun Shi

**Affiliations:** 1 Department of Hepatobiliary and Pancreatic Surgery, Qingdao Municipal Hospital Affiliated to Qingdao University, Qingdao, Shandong Province 266000 People’s Republic of China; 2 The Second Department of Gastrointestinal Surgery, Jinan Central Hospital Affiliated to Shandong University, No.105 Jiefang Road, Jinan, Shandong Province 250013 People’s Republic of China; 3 Computer Network Information Center, Chinese Academy of Sciences, Beijing, 100190 People’s Republic of China; 4 University of the Chinese Academy of Sciences, Beijing, 100190 People’s Republic of China; 5 ChosenMed Technology (Beijing) Co. Ltd, Beijing, 100176 People’s Republic of China

**Keywords:** Hepatocellular carcinoma, Biomarker, Prognostic, Immune

## Abstract

**Background:**

The mortality rate of hepatocellular carcinoma (HCC) remains high worldwide despite surgery and chemotherapy. Immunotherapy is a promising treatment for the rapidly expanding HCC spectrum. Therefore, it is necessary to further explore the immune-related characteristics of the tumour microenvironment (TME), which plays a vital role in tumour initiation and progression.

**Methods:**

In this research, 866 immune-related differentially expressed genes (DEGs) were identified by integrating the DEGs of samples from The Cancer Genome Atlas (TCGA)-HCC dataset and the immune-related genes from databases (InnateDB; ImmPort). Afterwards, 144 candidate prognostic genes were defined through weighted gene co-expression network analysis (WGCNA).

**Results:**

Seven immune-related prognostic DEGs were identified using the L1-penalized least absolute shrinkage and selection operator (LASSO) Cox proportional hazards (PH) model, and the ImmuneRiskScore model was constructed on this basis. The prognostic index of the ImmuneRiskScore model was then validated in the relevant dataset. Patients were divided into high- and low-risk groups according to the ImmuneRiskScore. Differences in the immune cell infiltration of patients with different ImmuneRiskScore values were clarified, and the correlation of immune cell infiltration with immunotherapy biomarkers was further explored.

**Conclusion:**

The ImmuneRiskScore of HCC could be a prognostic marker and can reflect the immune characteristics of the TME. Furthermore, it provides a potential biomarker for predicting the response to immunotherapy in HCC patients.

**Supplementary Information:**

The online version contains supplementary material available at 10.1186/s12885-021-08052-8.

## Background

Hepatocellular carcinoma (HCC) is one of the most common malignancies [1, 2]. With a 5-year survival rate of 18%, HCC is the second most lethal tumour after pancreatic cancer [3] and the fourth leading cause of cancer-related mortality worldwide [4, 5]. HCC is the main type of primary liver cancer, and its increasing mortality rate is receiving growing concern. However, conventional treatments such as radiotherapy and chemotherapy do not significantly prolong overall survival (OS) in HCC patients [6].

Immunotherapy with immune checkpoint inhibitors (ICIs) is also an important treatment option. These therapies are revolutionizing the clinical treatment pattern of multiple tumours, most notably advanced melanoma [7–13], non-small-cell lung cancer [14, 15] and renal cell carcinoma (RCC) [16, 17]. Since HCC benefits from programmed cell death protein 1 (PD-1) pathway blockade [18], approved ICIs may be used in alternative HCC treatment strategies in the near future [6, 19, 20]. Although significant progress has been achieved with ICIs, only a small number of patients can benefit from them [21]. Therefore, there is an urgent need for new, immune-based biomarkers to identify HCC patients who may have better prognoses after immunotherapy.

Immune cells play an important role in the HCC microenvironment and show clinicopathological significance in predicting prognosis and treatment efficacy [22–24]. The characteristics of the tumour microenvironment (TME) and their functional impact on immunotherapy are actively being studied.

**Fig. 1:**
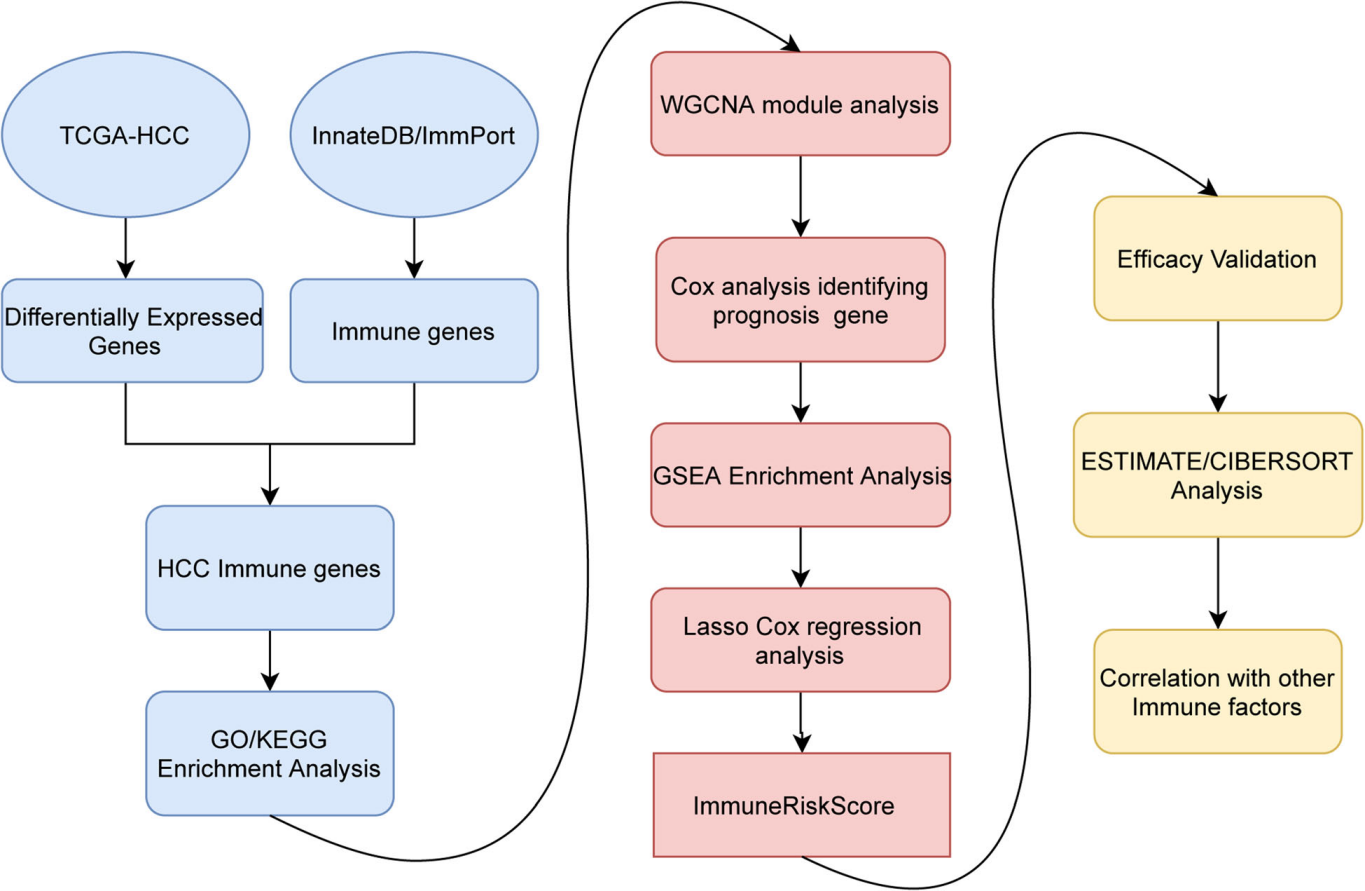
Overview of the experiments

In this study, we made full use of The Cancer Genome Atlas (TCGA) data and an a priori set of immune-related genes to construct a prognostic immune risk score using weighted gene co-expression network analysis (WGCNA) and the least absolute shrinkage and selection operator (LASSO) Cox model. We also analysed the correlation between the ImmuneRiskScore and the infiltration level of different immune cells to clarify potential mechanisms for the formation of the microenvironment. Finally, we explored its relevance to other immune biomarkers and its potential to identify patients eligible for immunotherapy to improve the therapeutic effects. The overall process is shown in Fig. 1.

## Methods

### Data download and processing

We downloaded the RNA sequencing (RNA-seq) expression profile (count and fragments per kilobase of transcript per million mapped reads (FPKM) format) and clinical data of the TCGA-HCC dataset from the University of California, Santa Cruz (UCSC) Xena data portal (https://xena.ucsc.edu/), which contains information on 50 normal samples and 374 tumour samples. An immune-related gene set containing 1052 immune genes was downloaded from InnateDB (https://www.innatedb.ca/) (Table S1), and a gene set containing 1811 immune-related genes was downloaded from ImmPort [25] (https://www.immport.org) (Table S2). Expression profiling data and clinical data were obtained from the GSE14520 by the GEOquery [26] package of Bioconductor in R-3.5.2. GSE14520 contains 162 tumour samples after removing the normal samples. The microarray probe IDs were mapped to gene symbols based on the GPL3921 platform (Affymetrix HT Human Genome U133A Array) and incorporated in the dataset matrix for each dataset. Eventually, the average of multiple probes that correspond to a single gene for each dataset was calculated individually using in-house R scripts. The tumour mutation burden (TMB) data was downloaded from the PanCancer Atlas (https://www.cell.com/consortium/pancanceratlas).

To further verify the predictive performance of the ImmuneRiskScore on immunotherapy, we collected the gene expression profiles and clinical response information of 33 RCC patients who received ICI therapies [27]. In addition, we collected the RNA-seq data of 65 melanoma patients treated with anti-PD-1 or anti-PD-L1 therapies [28, 29]. For each dataset, we standardized the transcriptome data across patients by max-min normalization.

### Differentially expression genes

Limma [30] packages in R-3.5.2 were employed to identify differentially expressed genes (DEGs) between tissue adjacent to cancerous (*n* = 50) and cancer (*n* = 367) patients based on the raw counts for HCC gene expression from the TCGA. The empirical Bayes method was applied in the limma package to select significant DEGs. Here, the standard comparison mode was employed, and the threshold was treated as *p*-value < 0.05 and |log2-fold change| > = 1.5.

### Gene ontology (GO) and pathway enrichment analysis of DEGs

In this research, the clusterProfiler package [31] was used to identify and visualize the GO terms (including biological process, cellular component, and molecular function terms) and Kyoto Encyclopedia of Genes and Genomes (KEGG) pathways enriched for DEGs. We set a p-value < 0.01 as the cut-off criterion and BH as the significant adjustment method; the cut-off for the q-value was also set as 0.01.

### WGCNA

The transcript FPKM data was used for WGCNA [32]. First, the original data was preprocessed to identify samples with missing data and exclude outliers. Second, the soft-thresholding power was selected with the pickSoftThreshold package, which can calculate the scaleless topological fitting index for several powers and provide the appropriate soft-thresholding power for network construction. Third, we performed one-step automatic network construction and module detection. Adjacent relationships were converted into topological overlaps to measure the network connectivity of a gene as the sum of its connectivity to its neighbours and to all other genes to generate a network. A hierarchical clustering function was used to divide genes with similar expression profiles into several modules [33]. Next, key modules related to OS were selected and visualized by Cytoscape [34]. In the present study, we calculated the correlation between module eigengenes and the clinical traits survival events and survival time to determine the relevant module. Then, through linear regression analysis of gene expression and clinical information, we defined gene significance (GS) as the log10 transformation of the *P*-value (GS = lgP). In a module, the module significance (MS) was defined as the average GS for all the genes. Hub genes were identified as those with high clinical trait significance (> 0.1) and high intramodular connectivity (> 0.5) in relevant modules, and they were selected as candidate genes for further analysis and validation.

### Gene set enrichment analysis of hub genes

Gprofiler2 (https://CRAN.R-project.org/package=gprofiler2) was used to perform overrepresentation analysis on input HCC hub genes [35]. It maps these immune genes to known sources of functional information and detects significantly enriched terms. We included pathways from KEGG (https://www.genome.jp/kegg/), Reactome (https://reactome.org/), and CORUM (http://mips.helmholtz-muenchen.de/corum/). This method can obtain *p*-values by the hypergeometric test and perform false discovery rate (FDR) correction for multiple testing.

### Construction and validation of an immune-related prognostic model

The univariate Cox proportional hazards (PH) regression model in the ‘survival’ package was used to calculate the hazard ratio (HR) for DEGs of the HCC cohort. DEGs with significance (*p*-values < 0.05) were analysed, and their survival risks were recalculated using the Cox regression model from the glmnet R package [35] so that the most important prognostic genes were selected. The regularization path of the LASSO method was calculated by setting the regularization parameter lambda to 1. To predict patient survival, the formula for the ImmuneRiskScore model was established as follows:

$$ \mathrm{ImmuneRiskScore}=\sum \left( Normalized\ expression\ value\ of\ gene\  Gi\times LASSO\  cox\  coefficient\ of\ gene\  Gi\ \right) $$

Subsequently, we obtained the ImmuneRiskScore of 367 TCGA HCC patients and determined the threshold for the high- and low-risk groups based on the average.

### Estimation of immune cell type fractions and ESTIMATE score

CIBERSORT [36] and LM22 reference gene expression matrices were employed to quantify the cell composition of different HCC samples. The normalized gene expression data were analysed using the CIBERSORT algorithm with 1000 permutations. Afterwards, immune and stromal scores were calculated with ESTIMATE, an algorithm that provides information on the abundance of these cell types in tumour tissues [37].

### Statistical analysis

The survival curves of patients stratified according to the expression of hub immune genes were generated via the Kaplan-Meier method, and the statistical significance of differences was determined by the log-rank test. Receiver operating characteristic (ROC) curves were applied to assess the sensitivity and specificity of survival prediction based on the ImmuneRiskScore, and the pROC package was utilized to quantify the area under the curve (AUC). The nonparametric Mann-Whitney-Wilcoxon test was used to compare the data from different groups, and Pearson’s chi-square test was performed to measure the level of significance for associations among variables. All statistical analyses were performed using R-3.5.2. All reported *p*-values were two-tailed, and *p* < 0.05 was considered to indicate statistical significance.

## Results

### Identification of immune-related DEGs

**Fig. 2:**
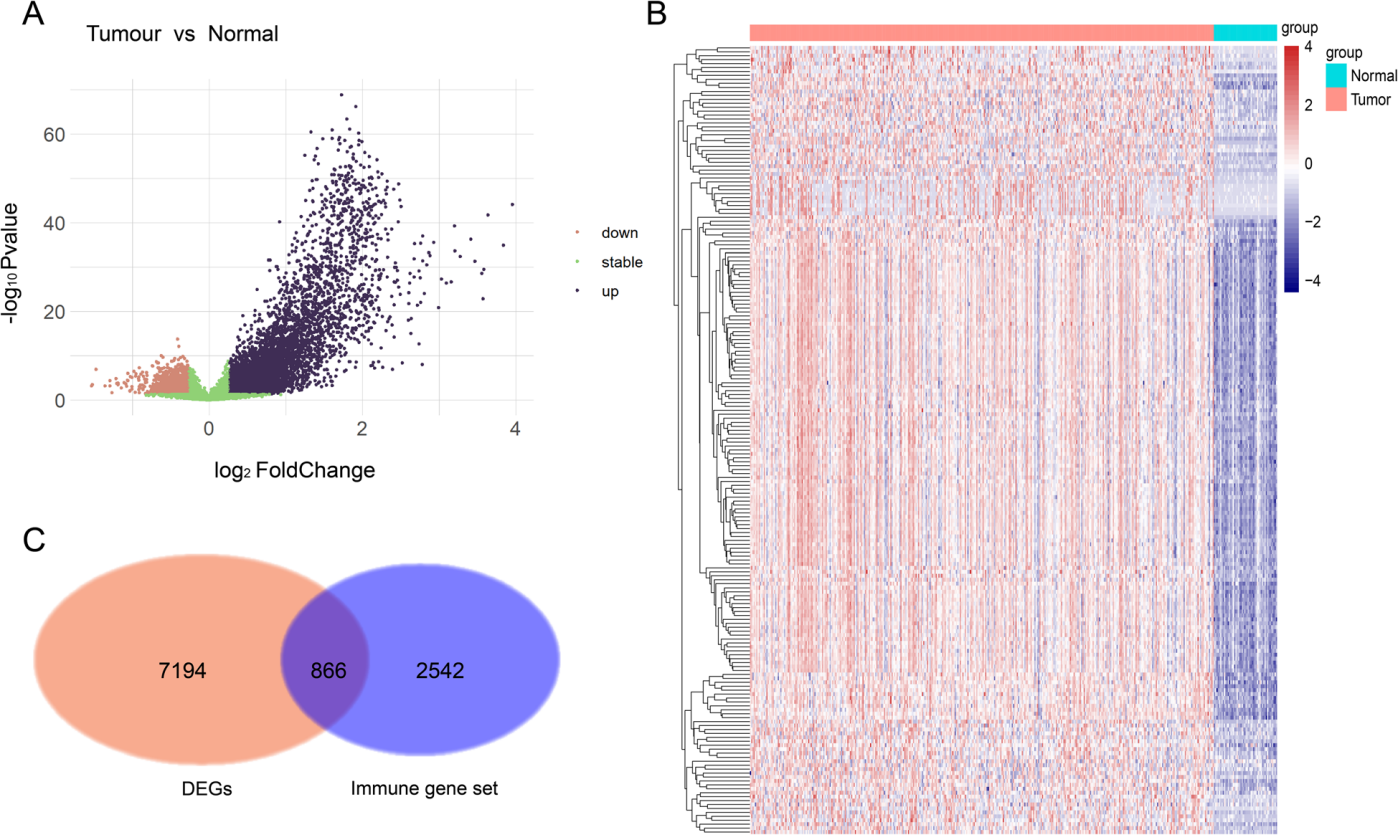
Identification of DEGs in 417 HCC patients. **a** Volcano plot of 7194 DEGs. The upper-left brown and upper-right blue dots represent genes that are down- and upregulated in HCC, respectively. **b** Unsupervised hierarchical clustering heatmap for the top 200 DEGs ranked by fold change. Red: upregulated DEGs; navy blue: downregulated DEGs. **c** Venn diagram of immune genes and DEGs

From the TCGA database, we obtained the expression profiles of 417 HCC samples, including 367 tumour samples and 50 normal samples after data preprocessing. A total of 7194 genes were identified as DEGs with the threshold of p-value < 0.05 and |log2-fold change| > 1.5; of the genes, 3657 were upregulated and 3537 were downregulated (Fig. 2a, Table S3). The samples were well clustered into normal and tumour groups when the top 200 DEGs were selected for unsupervised hierarchical clustering (Fig. 2b). To obtain the immune-related DEGs for HCC samples, we had to select genes that were both immune-related and differentially expressed in the two groups. Thus, we collected 2542 human immune-related genes (1811 immune-related genes from ImmPort and 1052 innate immune-related genes from InnateDB). We overlapped the DEGs with immune-related genes and selected 866 immune-related DEGs (Fig. 2c, Table S4) for further analysis.

**Fig. 3:**
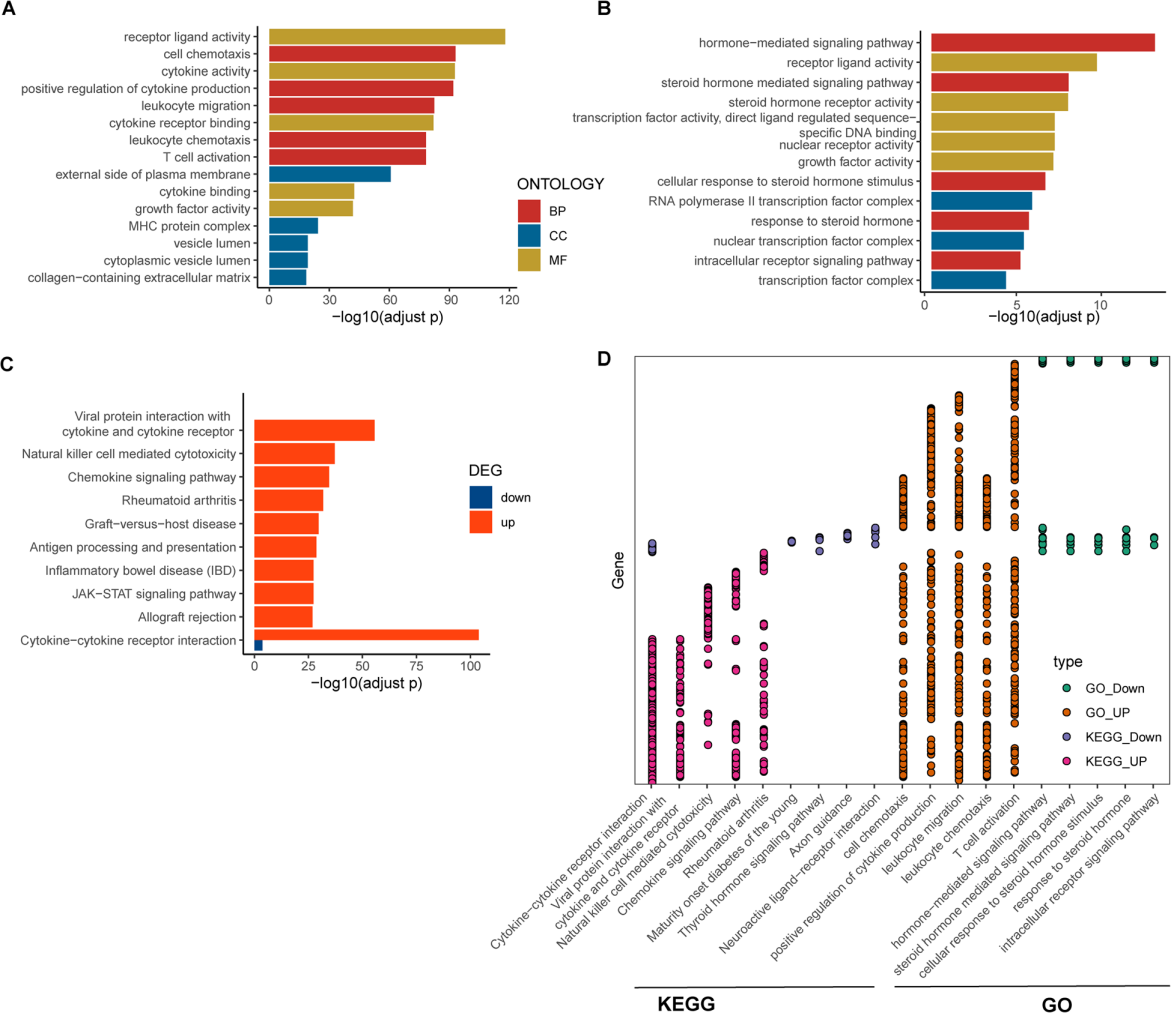
Enrichment analysis of the immune-related DEGs. **a** GO results of upregulated immune-related DEGs. **b** GO results of downregulated immune-related DEGs. **c** KEGG analysis of immune-related DEGs. Red bars represent upregulated DEGs, and blue bars represent downregulated DEGs. **d** DEGs (y axis) significantly enriched in GO terms and KEGG pathways (x axis). Pink: GO terms enriched for the upregulated DEGs; purple: GO terms enriched for the downregulated DEGs; orange: KEGG pathways enriched for the upregulated DEGs; green: KEGG pathways enriched for the downregulated DEGs

The results of GO term enrichment analysis varied according to GO classification and differences in the expression of immune-related DEGs. In terms of biological processes, the upregulated genes were significantly enriched in cell chemotaxis, positive regulation of cytokine production, leukocyte migration, etc., and the downregulated genes were enriched in the hormone-mediated signalling pathway, the steroid hormone mediated signalling pathway, etc. Regarding cellular components, upregulated immune-related DEGs were significantly enriched on the external side of the plasma membrane, major histocompatibility complex (MHC) protein complex, cytoplasmic vesicle lumen, vesicle lumen etc., and the downregulated DEGs were significantly enriched in the RNA polymerase II transcription factor complex, nuclear transcription factor complex, and transcription factor complex. In terms of molecular function, the upregulated immune-related DEGs were significantly enriched in receptor ligand activity, cytokine activity, etc. and the downregulated DEGs were significantly enriched in receptor ligand activity, steroid hormone receptor activity, and nuclear receptor activity. More detailed GO enrichment analysis results are shown in Fig. 3a, b and Table S5. These significantly enriched pathways and terms help us better understand the role of DEGs in the HCC immune microenvironment.

Ten significantly enriched KEGG pathways for the upregulated genes are shown in Fig. 3c and Table S6: cytokine-cytokine receptor interaction, viral protein interaction with cytokine and cytokine receptor, natural killer (NK) cell-mediated cytotoxicity, the chemokine signalling pathway and rheumatoid arthritis. The top 10 KEGG and GO enrichment results for the up- and downregulated DEGs are detailed in Fig. 3d, and most of the genes enriched in the top five KEGG pathways and GO terms were upregulated genes. As shown in Fig. 3d, 168 upregulated DEGs and 11 downregulated DEGs were enriched in cytokine-cytokine receptor interactions, which indicated that there may be a complicated molecular mechanism in the HCC immune microenvironment.

### Weighted co-expression network construction and key modules identification

**Fig. 4:**
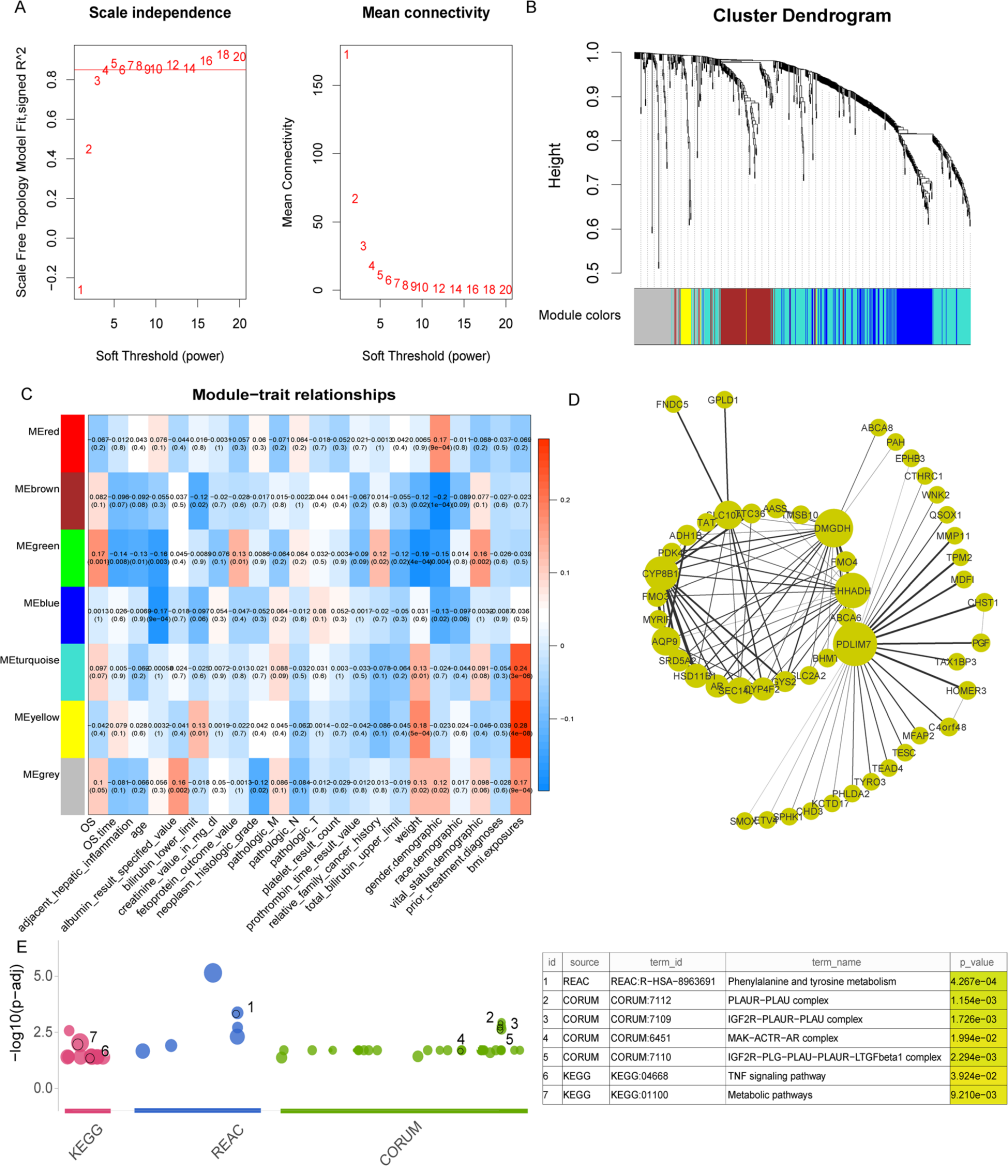
WGCNA and enrichment analysis. **a** Scale-free fit index analysis for various soft-thresholding powers, cut-off > 0.85. The mean connectivity analysis for various soft-thresholding powers. **b** The cluster dendrogram of DEGs. In the figure, each branch represents one gene, and each colour indicates a co-expression module. The grey refers to genes that cannot be classified in any module. **c** Heatmap of the correlation between module eigengenes and the clinical traits of HCC. The green module was most relevant to the OS events and OS time. **d** The top 50 hub genes in the green module. The genes are represented as nodes; node size is related to connectivity of the gene by degree, and edge size is related to weight. **e** Enrichment analysis of immune hub genes. Left: The x axis represents the functional terms that are grouped and colour-coded according to data sources. The y-axis indicates the adjusted *p*-values on a negative-log10 scale. Every circle is one term and is sized according to the term enrichment degree. Right: the table of interesting enrichment results

The 866 immune-related DEGs from 367 HCC tumour samples were used to construct the gene co-expression network. After handling the missing values, we detected the outlier samples by hierarchical clustering (Figure S1), and the dendrogram showed the 5 outlier samples that were removed from the analysis.

performed network topology analysis for thresholding powers from 1 to 20, and 4 was the lowest power with a scale-free topology fit index of 0.85 (Fig. 4a). We obtained a gene clustering tree using hierarchical clustering of topological overlap measure (TOM)-based dissimilarity and identified 6 modules (Fig. 4b, Table 1). To select the clinically significant modules, we used WGCNA to calculate the correlations between the external clinical information and gene modules. As shown in Fig. 4c, the green module was the most associated with OS, and the green and brown eigengenes were highly relevant (Figure S2).

**Table 1:** The number of genes in different modules

Module	The number of genes
Blue	302
Brown	147
Green	139
Red	86
Turquoise	389
Yellow	139
Grey	254

We visualized the green module as a network by Cytoscape and selected the top 50 gene pairs by sorting the weight of gene pairs. As shown in Fig. 4d, some genes, such as *PDLIM7*, *EHHADH*, *DMGDH*, and *CYP8B1* are represented with larger circles indicating higher node degrees. Finally, we screened 144 immune hub genes with high significance in terms of OS events and OS time (> 0.1) and high relevance to the green module (> 0.5) (Table S7).

Gene set enrichment analysis was performed on 144 immune hub genes to find overrepresented functions in the context of biological pathways, such as those in the KEGG and Reactome databases, and in the context of complexes in CORUM. The results showed that the immune hub genes were significantly enriched in 52 pathways and complexes (*p* < 0.05) (Fig. 4e; Table S8), such as the *TNF* signalling pathway, tyrosine metabolism, *IGF2R − PLAUR − PLAU* complex, etc. These findings suggest that these hub genes not only affect the metabolism, apoptosis, cell survival, inflammation and immunity of HCC but also play a pivotal role in regulating the protein complexes of immune cells.

### Construction of a prognostic gene signature with the LASSO Cox PH model

**Fig. 5:**
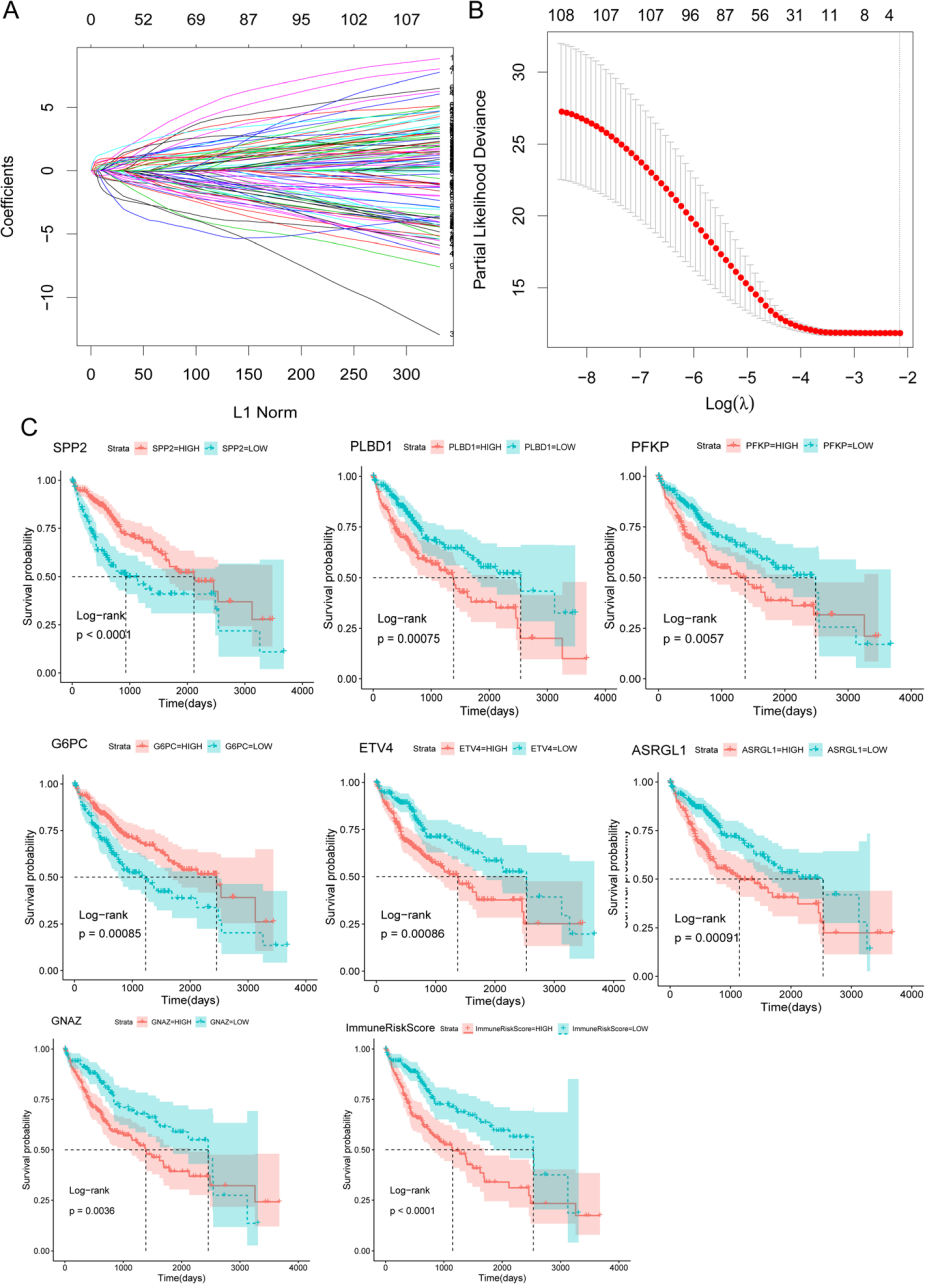
LASSO Cox regression analysis of immune-related genes in HCC. **a** The solution path plot of each independent variate. The lateral axis and longitudinal axis represent the lambda value and independent variable coefficient, respectively. Each curve corresponds to a variable. **b** The confidence interval for each lambda, including the cross-validation curve (red dotted line) and upper and lower standard deviation curves along the λ sequence (error bars). The selected λ is shown by the vertical dotted lines. LASSO, least absolute shrinkage and selection operator. **c** Analysis of the prognostic value of the selected LASSO cox hub genes and ImmuneRiskScore

We revealed that 108 of the 144 immune hub genes were significantly associated with OS through univariate Cox regression analysis (Table S9). Subsequently, LASSO-penalized Cox analysis was performed to further narrow the scope of OS-related hub genes (Fig. 5a and b). As a result, seven genes were identified to construct the ImmuneRiskScore model for evaluating the prognosis of HCC patients. The details of the seven genes and their Cox coefficients are listed in Table 2. GO enrichment analysis showed that these seven genes were enriched in several molecular functions: asparaginase activity, beta-aspartyl-peptidase activity, 6-phosphofructokinase activity, glucose-6-phosphatase activity, and sugar-terminal-phosphatase activity.

**Table 2:** The result of LASSO regression

Genes	Coef.
*Secreted Phosphoprotein 2 (SPP2)*	−0.27112
*Glucose-6-Phosphatase Catalytic Subunit (G6PC)*	−0.12328
*Phospholipase B Domain Containing 1 (PLBD1)*	0.66909
*ETS Variant Transcription Factor 4 (ETV4)*	0.359013
*Phosphofructokinase Platelet (PFKP)*	0.402211
*G Protein Subunit Alpha Z (GNAZ)*	0.126409
*Asparaginase And Isoaspartyl Peptidase 1 (ASRGL1)*	0.795796

Next, HCC patients were divided into high- and low-score groups based on the LASSO Cox model-identified hub genes and ImmuneRiskScore, and the optimal threshold was obtained from the survminer package. The results indicated that five genes (*PLBD1, ETV4, PFKP, GNAZ, ASRGL1*) were risk factors, while *SPP2* and *G6PC* were protective factors (Fig. 5c). In addition, high-scoring samples had worse OS than low-scoring samples. The prognostic accuracy of the ImmuneRiskScore (95% confidence interval (CI) for the HR: 0.48 (0.33–0.68), log-rank test *p* < 0.0001) is shown in the last figure of Fig. 5c. Additionally, the results of multivariate Cox regression analyses showed that the predictive value of the ImmuneRiskScore was independent of common clinical variables (Table S10).

### Verification of the ImmuneRiskScore in another HCC cohort

**Fig. 6:**
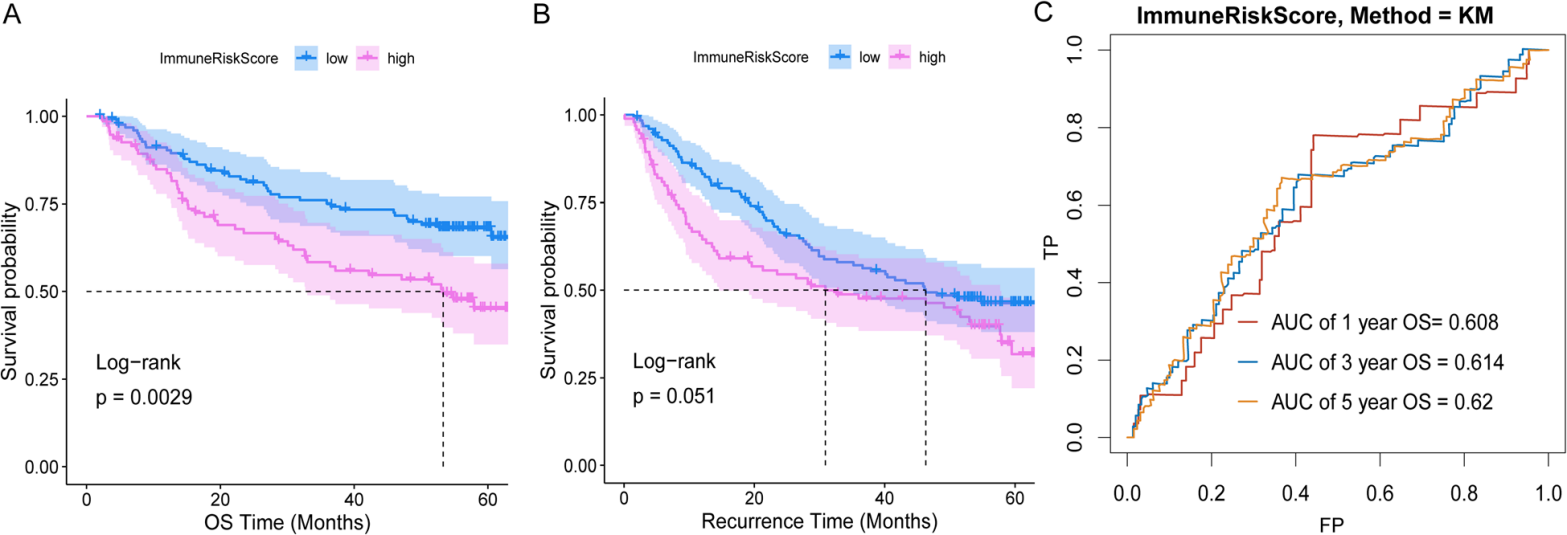
Validation of the ImmuneRiskScore model in the HCC cohort. **a** The OS of the high-risk group was significantly shorter than that of the low-risk group. **b** The recurrence rate of the high-risk group was higher than that of the low-risk group. **c** Time-dependent ROC curve analysis of the ImmuneRiskScore

To further investigate the prognostic value of the ImmuneRiskScore, we conducted a validation analysis in another Gene Expression Omnibus (GEO) cohort (GSE14520, *n* = 221). The samples were categorized into two groups based on the ImmuneRiskScore, and the results indicated the significant prognostic value of the ImmuneRiskScore in predicting OS as well as recurrence (Fig. 6a and b). Figure 6c shows the prognostic accuracy of the ImmuneRiskScore, which was considered a continuous variable in this experiment. The AUC of the ROC curve of the prognostic model for OS was 0.608 at 1 year, 0.614 at 3 years, and 0.620 at 5 years. These results suggest that the ImmuneRiskScore could be a potential survival predictor.

### Landscape of stromal and immune cell infiltration in patients with high and low ImmuneRiskScore values

**Fig. 7:**
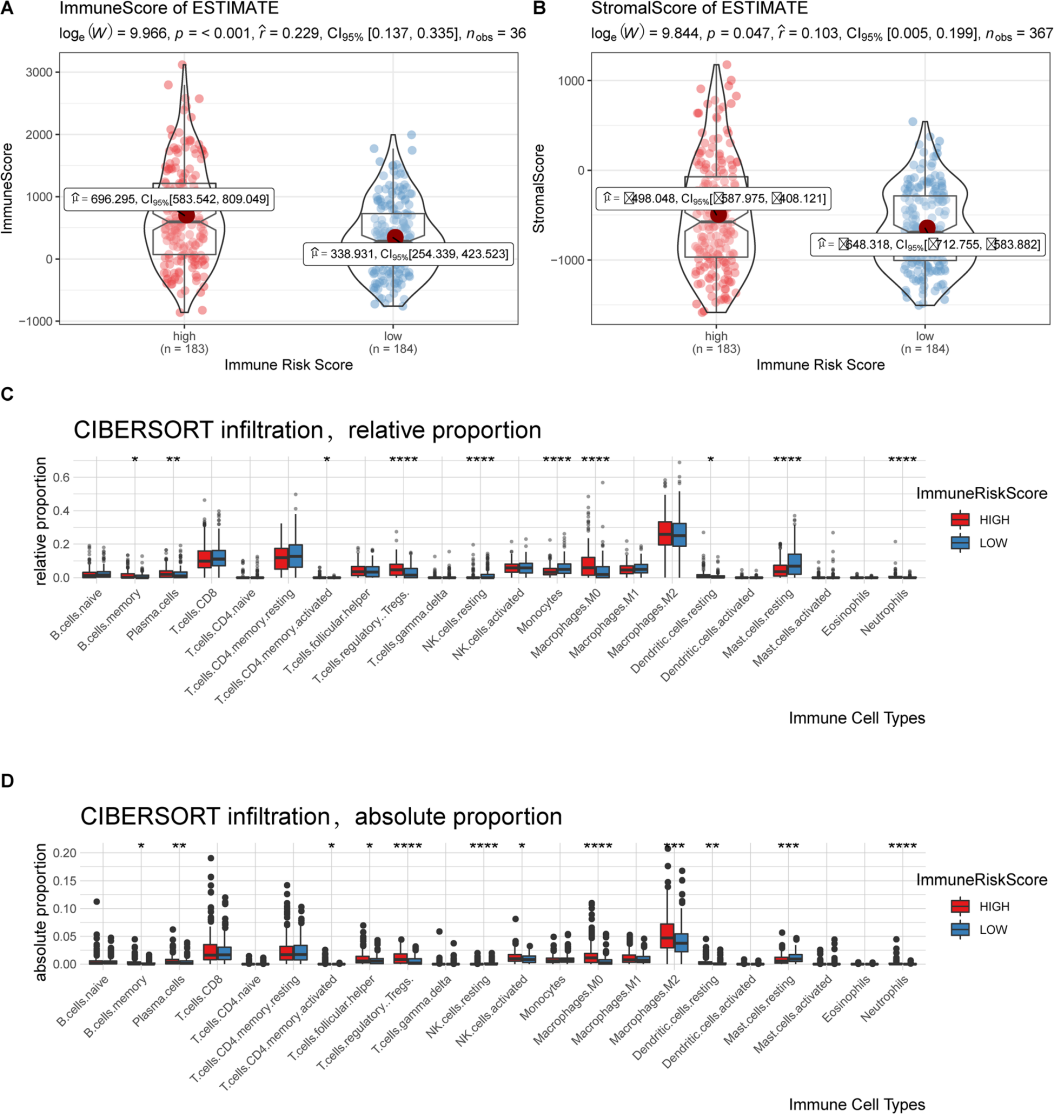
Profile of the immune microenvironment between the low- and high-ImmuneRiskScore groups. **a** Difference in the distribution of immune score values in the low- and high-ImmuneRiskScore groups. **b** Difference in the distribution of stromal score values in the low- and high-ImmuneRiskScore groups. **c** The relative proportion of immune cell categories in the low- and high-ImmuneRiskScore groups. **d** The absolute proportion of immune cell categories in the low- and high-ImmuneRiskScore groups. Comparisons between the two groups were performed through the Wilcoxon rank-sum test. Each boxplot is labelled with asterisks indicating the *p*-values (*p < 0.05, **p < 0.01, ***p < 0.001, ****p < 0.0001)

The infiltrating cells and tumour purity of tumour tissues were assessed by ESTIMATE [37]. The stromal score represented the presence of stromal cells in tumour tissue, and the immune score indicated the infiltration of immune cells in tumour tissue. Both of them were used for the determination of tumour purity (Table S11). We then showed differences in terms of stromal and immune scores in high-risk and low-risk HCC patients (Fig. 7a and b).

Both the immune score and stromal score of high-risk patients were significantly higher than those of low-risk patients (Wilcox, *p* < 0.05), indicating that immune and stromal cell infiltration were associated with the ImmuneRiskScore.

We further estimated the proportions of 22 immune cell types in HCC patients using CIBERSORT [36] (Table S12). We compared the relative proportions of 22 immune cell types between patients with low and high ImmuneRiskScore values and found significant differences in memory B cells, plasma cells, CD4 memory activated T cells, regulatory T cells (Tregs), etc. (Fig. 7c).

Furthermore, we calculated the absolute immune infiltration score of these 22 immune cell types in combination with tumour purity. Afterwards, a similar comparison of absolute proportions of these 22 immune cell types was made between low and high ImmuneRiskScore patients. Significant differences were found in the proportions of memory B cells, plasma cells, CD4 memory-activated T cells, etc. (Fig. 7c).

Both the relative and absolute proportions of several immune cell types, including memory B cells, plasma cells, CD4 memory-activated T cells, Tregs, resting NK cells, M0 macrophages, resting dendritic cells, resting mast cells, and neutrophils, were associated with the ImmuneRiskScore. The results also demonstrate that the changes in the proportions of immune cells may be indirectly associated with the OS of HCC patients.

### Correlations between the ImmuneRiskScore and immune biomarkers

It is well documented that immune biomarkers in the TME can effectively predict the clinical benefit of ICIs, which are revolutionizing the clinical treatment landscape. We next introduced a few important immune biomarkers, including PD-L1, PD-1, PD-L2, CTLA4, cytolytic activity (CYT), and interferon-gamma (IFN-γ). Among these biomarkers, the immune checkpoint genes PD-L1, PD-1, PD-L2, and CTLA4 are co-expressed in HCC [38]. The CYT value reflects the activity of cytotoxic T cells (CTLs) and NK cells due to their powerful ability to lyse tumour cells [39]. A recent study found that CYT-high HCC has stronger immunogenicity and a more favourable TME than CYT-low HCC, which would result in better clinical outcomes [40]. CYT is measured based on the geometric mean of expression of granzyme A (GZMA) and perforin (PRF1). IFN-γ is a key cytokine that activates the PD-1 signalling axis by directly upregulating the ligands PD-L1 and PD-L2 in tumour cells produced by activated T cells, NK cells and NK T cells [41, 42]. The expression of the IFN-γ receptor can affect the mechanism of escape from host immune surveillance in HCC [43].

TMB is defined as the number of nonsynonymous mutations per megabase sequenced. A high TMB is associated with an improved response to immune checkpoint blockade in HCC [44], melanoma [45], and non-small-cell lung cancer [46, 47]. Dysregulation of the transforming growth factor beta (TGF-β) pathway plays a central role in inflammation, fibrogenesis, and immunomodulation in the HCC microenvironment [48, 49]. TGF-β signalling in fibroblasts is documented as a pleiotropic cytokine associated with poor prognosis in multiple tumour categories [50, 51] and is considered critical in advanced cancers in terms of the promotion of immunosuppression, angiogenesis, metastasis, tumour cell epithelial to mesenchymal transition (EMT), fibroblast activation and desmoplasia [52–54].

**Fig. 8:**
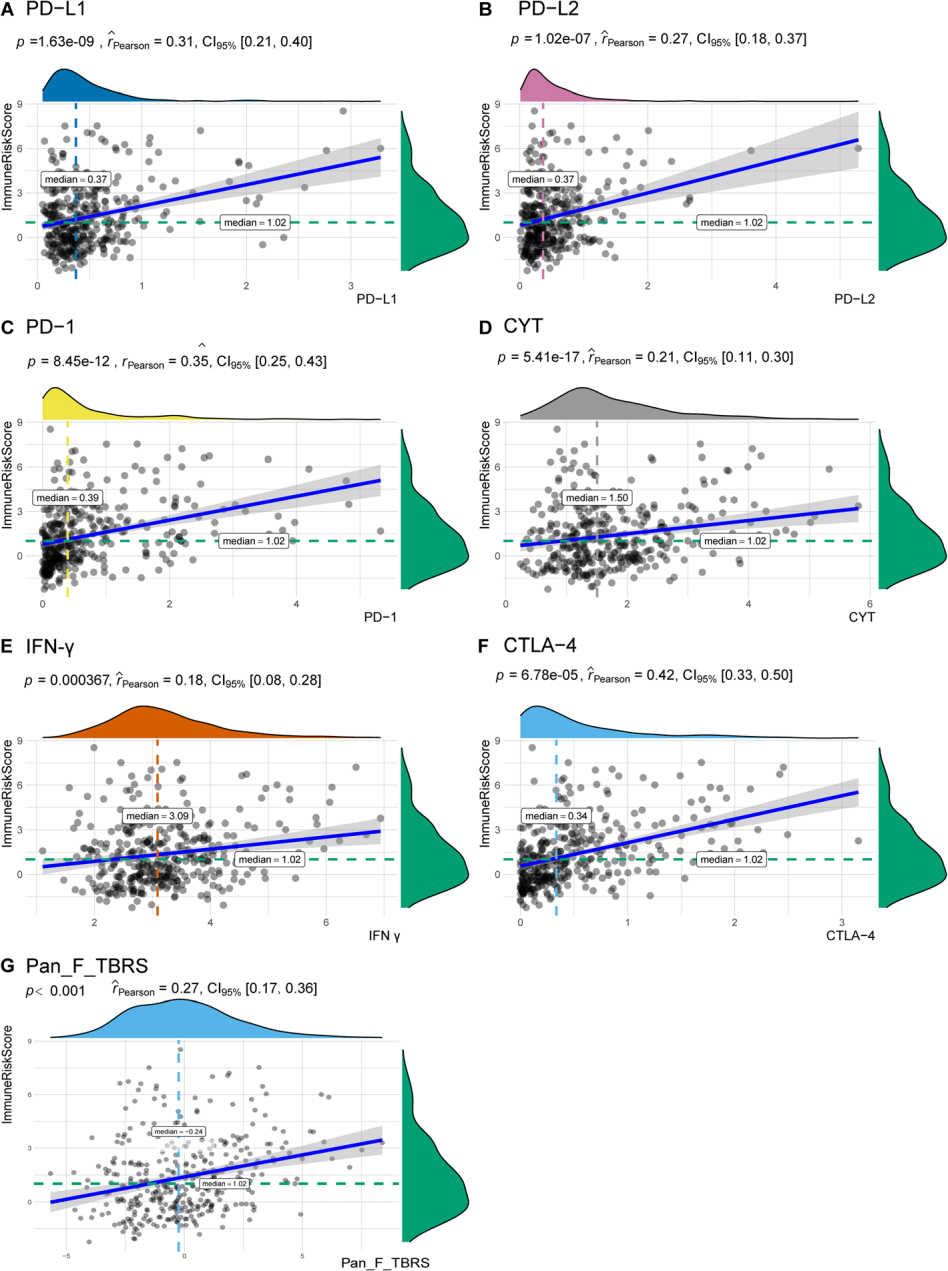
Correlation scatterplots between the ImmuneRiskScore and combined with a density plot of expression distribution. The ICB biomarkers included PD-L1, PD-1, PD-L2, CTLA-4, CYT, IFN-γ, and Pan-F-TBRS

We further explored the relationship between the ImmuneRiskScore and these immune biomarkers (Table S13, Table S14), which satisfied a bivariate normal distribution (Pearson result in Table S15). As shown in Fig. 8, the ImmuneRiskScore values were significantly positively related to several immune inflammation biomarkers (PD-L1: *r* = 0.31; *p* = 1.63e-09, 95% CI: 0.21–0.40; PD-1: *r* = 0.35; *p* = 8.45e-12, 95% CI: 0.25–0.43; PD-L2: *r* = 0.27; *p* = 1.02e-07, 95% CI: 0.18–0.37; CTLA-4: *r* = 0.42; *p* = 6.78e-05, 95% CI: 0.33–0.50; CYT: *r* = 0.21; *p* = 5.41e-17, 95% CI: 0.11–0.30, IFN-γ: *r* = 0.42; *p* = 0.00037, 95% CI: 0.17–0.36) and Pan-F-TBRS (*r* = 0.27; *p* = 2.51e-07, 95% CI: 0.33–0.50). The *p*-values of all these correlations were smaller than 0.001, suggesting that the ImmuneRiskScore is correlated with immune biomarkers. In addition, there was no correlation between the ImmuneRiskScore and TMB, indicating that the TMB is an independent factor mediating the TME in these two groups.

**Fig. 9:**
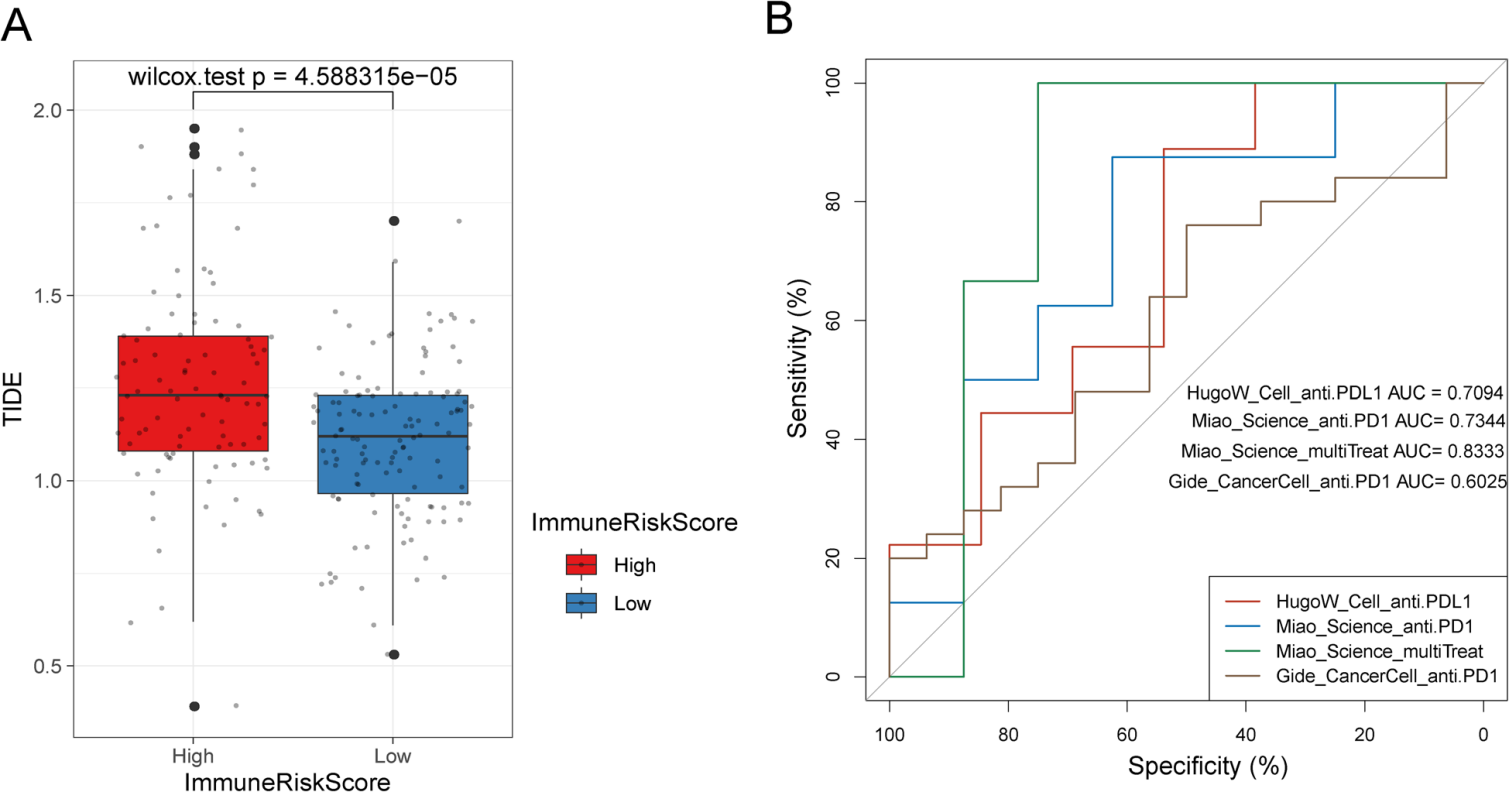
The value of the ImmuneRiskScore for predicting the response to ICB treatment. **a** Correlation between TIDE and ImmuneRiskScore (*p* < 0.0001, Wilcoxon test). **b** The performance of the ImmuneRiskScore in predicting ICB response in four datasets

Tumour immune dysfunction and exclusion (TIDE) is a gene expression biomarker developed for predicting the clinical response to immune checkpoint blockade (ICB) therapy [55]. We obtained the TIDE score for the GSE14520 dataset (Table S16) through an online webserver (http://tide.dfci.harvard.edu/). There was a significant difference in TIDE scores between the high and low ImmuneRiskScore values (Wilcox test *p* = 4.588315e-05) (Fig. 9a).

We further tested the predictive performance of the ImmuneRiskScore on the efficacy of ICB treatment in four public RNA-seq datasets (melanoma: HugoW_Cell_anti.PD-L1/Gide_CancerCell_anti.PD-1; RCC: Miao_Science_anti.PD-1/Miao_Science_multiTreat). The ImmuneRiskScore model achieved AUCs of 0.71 and 0.60 in predicting the response of melanoma patients treated with anti-PD-1 and anti-PD-L1 antibodies, respectively. It also achieved AUCs of 0.73 and 0.83 in predicting the response of RCC patients treated with anti-PD-1 and anti-CTLA4 antibodies, respectively (Fig. 9b). These results indicate that the ImmuneRiskScore might be a potential biomarker for predicting the immunotherapy response.

## Discussion

Increasing evidence indicates that immune-related biomarkers are associated with the prognosis of various cancer types [56–58]. However, biomarkers that can be used directly to determine the efficacy of cancer immunotherapy and the prognosis of patients remain to be explored.

Therefore, we propose the ImmuneRiskScore, which can predict OS based on the TME of HCC. We performed WGCNA, and identified 6 modules and found that the green module was highly relevant to the OS events and OS time. To identify clinically significant hub genes in the green module, we then performed LASSO Cox regression analysis and finally screened 7 genes: *SPP2, G6PC, PLBD1, ETV4, PFKP, GNAZ,* and *ASRGL1*. Then, a seven-gene risk scoring system was constructed, and this system successfully classified 417 HCC patients into two risk groups with significantly different survival rates. The predictive performance of the risk scoring model was successfully validated in an independent set from the GEO. This suggests that the ImmuneRiskScore based on these seven genes may be a promising prognostic biomarker and play an important role in the TME of HCC.

The pipeline constituted by WGCNA and LASSO Cox regression analysis is effective for many cancer types, such as glioblastoma [59], prostate [60], gastric cancer [61], lung cancer [62] and bladder cancer [63]. To the best of our knowledge, only two studies [64, 65] have used this pipeline to study HCC, but these studies did not focus on the immune microenvironment.

Generally, the ImmuneRiskScore is primarily a reflection of the constituents of the TME and accounts for the complex interactions between cancer cells, stromal cells, and immune cells. Thus, we aimed to further explore the relationship between these components. We found that the relative or absolute infiltration levels of memory B cells, plasma cells, activated CD4 memory T cells, Tregs, resting NK cells, M0 macrophages, resting dendritic cells, resting mast cells, and neutrophils were significantly associated with the ImmuneRiskScore, which also indicated the prognostic value of assessing these cell types. The infiltration of macrophages in solid tumours is associated with a poor prognosis and chemotherapy resistance in most cancers [66]. It is noteworthy that these cell types did not cover most of the T cell compartment components, which are a key part of the clinical response. In fact, other immune cells may also contribute to antitumour immunity [67–69]. For example, memory B cells also have a potential role in the response to ICB treatment [70].

To validate the potential clinical benefits of assessing the ImmuneRiskScore to guide ICI strategies, we explored the status of active innate and adaptive immune responses within the TME by gene expression profiling. The predictive value of our immune relation score was positively associated with PD-L1, PD-1, PD-L2, CTLA-4, CYT, IFN-γ and Pan-F-TBRS. These biomarkers are proinflammatory cytokine-related components of the inflammatory microenvironment of tumours [71, 72] and the TGF-β signalling pathway-related immune-excluded microenvironment of tumours [49].

Inflamed tumours contain proinflammatory cytokines and a type-I IFN signature, indicating activation of the innate immune response. TGF-β can drive the immune-excluded phenotype in the TME because it influences stromal cells and prevents T cells from penetrating into the tumour centre [49]. These results indicate that antitumour immunity is a bidirectional and dynamic system in the TME. This biomarker analysis will help to unravel the complexities of the interaction and molecular mechanisms between cancer and the host immune system.

We validated the predicted value of the ImmuneRiskScore by comparing it with that of the TIDE score. The results showed that the ImmuneRiskScore could possibly predict ICB clinical response based on pre-treatment tumour profiles. In addition, the predictive performance of the ImmuneRiskScore on four ICB treated cohorts indicate that it may be a potential immune-related biomarker for pan-cancer.

## Conclusions

In summary, we presented comprehensive insight into TME of HCC and identified ImmuneRiskScore, a potential biomarker that can be used to predict the response of immunotherapy for HCC patients, even for pan-cancer patients.

## Supplementary Information

**Additional file 1: Figure S1.** a: Clustering dendrogram of samples based on their Euclidean distance. b: Hierarchical clustering dendrogram of module eigengenes (top) and module heatmaps (bottom).

**Additional file 2: Figure S2.** Stacked bar charts of the infiltrating immune cells in HCC samples separated by high and low ImmuneRiskScore values.

**Additional file 3: Supplementary Table S1.** Genes downloaded from InnateDB.

**Additional file 4: Supplementary Table S2**. Genes downloaded from ImmPort.

**Additional file 5: Supplementary Tables S3.** Results of the differential expression analysis..

**Additional file 6: Supplementary Tables S4.** Immune-related differentially expressed genes (DEGs).

**Additional file 7: Supplementary Tables S5**. Results of the GO analysis.

**Additional file 8: Supplementary Tables S6.** Results of the KEGG analysis.

**Additional file 9: Supplementary Tables S7.** Immune-related hub genes related to OS.

**Additional file 10: Supplementary Tables S8.** Enrichment results for the 144 immune-related hub genes.

**Additional file 11: Supplementary Tables S9.** Results of the univariate Cox regression analysis.

**Additional file 12: Supplementary Tables S10.** Results of the multivariate Cox regression analysis.

**Additional file 13: Supplementary Tables S11.** ESTIMATE results for the TCGA-HCC dataset.

**Additional file 14: Supplementary Tables S12**. CIBERSORT results for the TCGA-HCC dataset.

**Additional file 15: Supplementary Tables S13.** Biomarkers in the TCGA-HCC dataset.

**Additional file 16: Supplementary Tables S14.** TMB data for the TCGA-HCC cohort.

**Additional file 17: Supplementary Tables S15.** Results for the Pearson analysis of biomarkers.

**Additional file 18: Supplementary Tables S16.** TIDE results for GSE14520.

## Data Availability

The datasets generated and analysed in the current study are available in the TCGA (https://xena.ucsc.edu/); in the GEO repository (https://www.ncbi.nlm.nih.gov/geo/) with the dataset numbers GSE14520 (https://www.ncbi.nlm.nih.gov/geo/query/acc.cgi?acc=GSE14520) and GSE78220 (https://www.ncbi.nlm.nih.gov/geo/query/acc.cgi?acc=GSE78220); in the European Nucleotide Archive (ENA) with the accession number PRJEB23709 (https://www.ebi.ac.uk/ena/browser/view/PRJEB23709); and in the Sequence Read Archive (SRA) (https://www.ncbi.nlm.nih.gov/sra) with the accession numbers SRP067938 and SRP090294.

## Abbreviations

GEO: Gene Expression Omnibus
TCGA: The Cancer Genome Atlas
HCC: Hepatocellular carcinoma
TME: Tumour microenvironment
TMB: Tumour mutation burden
DEGs: Differentially expressed genes
KEGG: Kyoto Encyclopedia of Genes and Genomes
GO: Gene ontology
PD-L1: Programmed death-ligand 1
PD-1: Programmed cell death protein 1
PD-L2: Programmed cell death 1 ligand 2
CTLA-4: Cytotoxic T-lymphocyte-associated protein 4
CYT: Cytolytic activity
IFN: Interferon
TGF-β: Transforming growth factor β
ICIs: Immune checkpoint inhibitors
ICB: Immune checkpoint blockade
WGCNA: Weighted gene co-expression network analysis
LASSO Cox PH model: L1-penalized least absolute shrinkage and selection. Operator Cox proportional hazards model
OS: Overall survival
