# Supplementary material for: Comprehensive analysis of immune-related prognostic genes in the tumour microenvironment of hepatocellular carcinoma
Source: BMC Cancer. 2021 Mar 31;21:331. doi: 10.1186/s12885-021-08052-8 (PMC8011181; doi:10.1186/s12885-021-08052-8)
Supplement: Supplementary file 1 — Additional file 1: Figure S1. a: Clustering dendrogram of samples based on their Euclidean distance. b: Hierarchical clustering dendrogram of module eigengenes (top) and module heatmaps (bottom). [file 12885_2021_8052_MOESM1_ESM.docx]

***
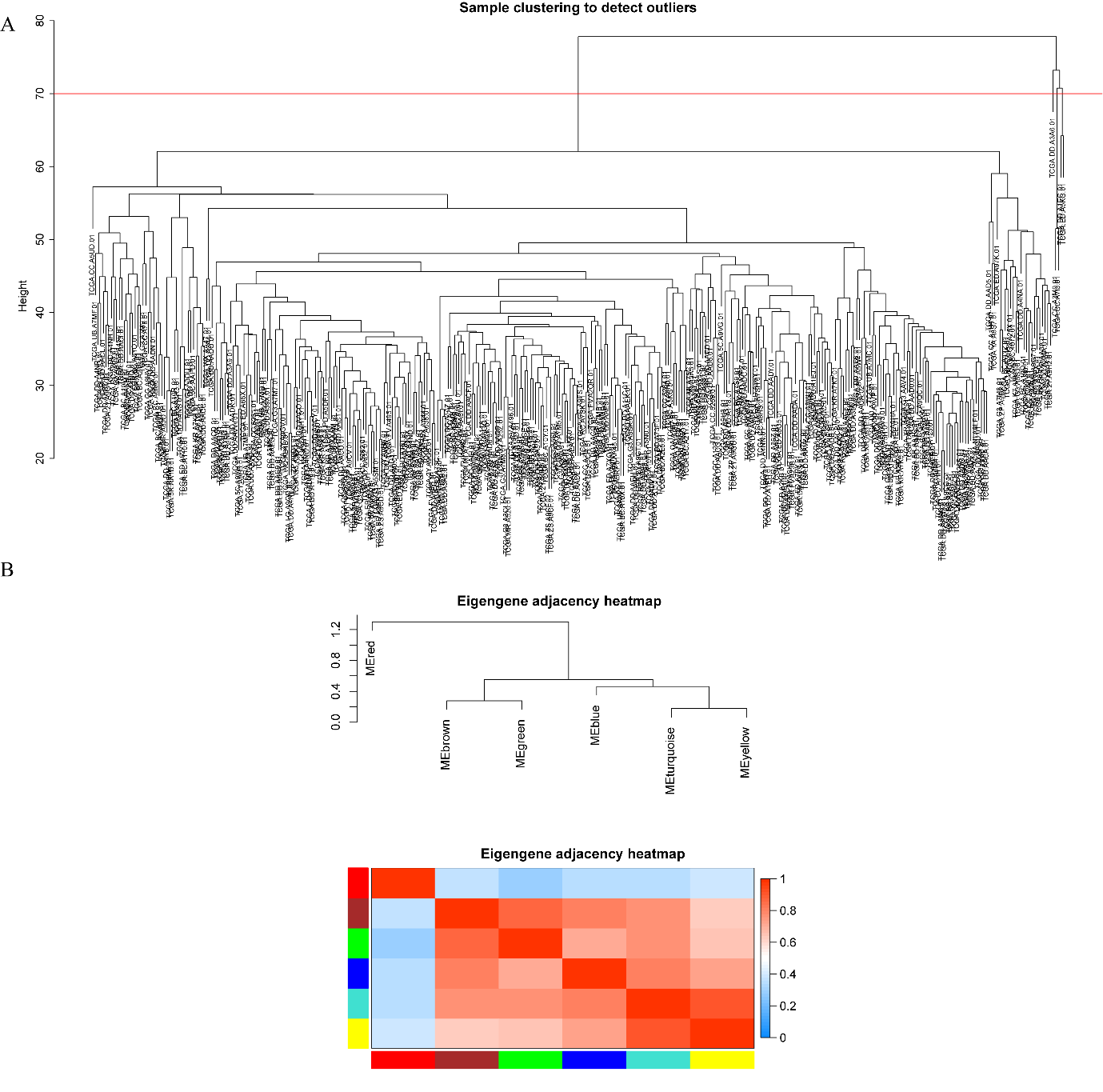
***

**Figure S1**. a: Clustering dendrogram of samples based on their Euclidean distance. b: Hierarchical clustering dendrogram of module eigengenes (top) and module heatmaps (bottom).
