# Supplementary material for: Comprehensive analysis of immune-related prognostic genes in the tumour microenvironment of hepatocellular carcinoma
Source: BMC Cancer. 2021 Mar 31;21:331. doi: 10.1186/s12885-021-08052-8 (PMC8011181; doi:10.1186/s12885-021-08052-8)
Supplement: Supplementary file 2 — Additional file 2: Figure S2. Stacked bar charts of the infiltrating immune cells in HCC samples separated by high and low ImmuneRiskScore values. [file 12885_2021_8052_MOESM2_ESM.docx]

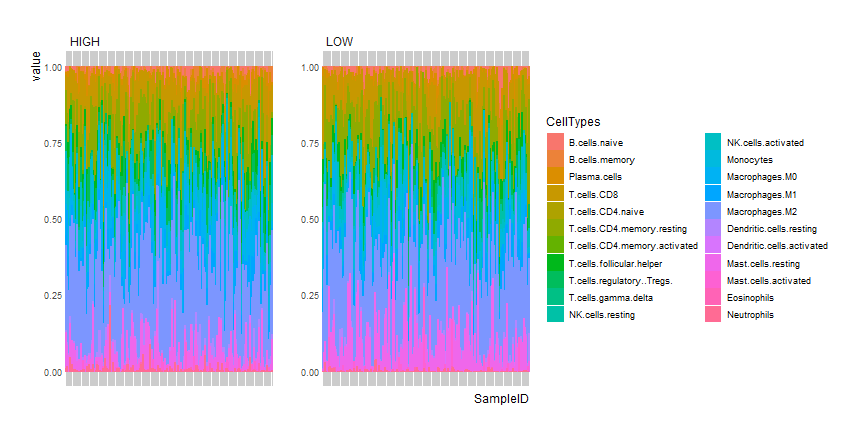


**Figure S2.** Stacked bar charts of the infiltrating immune cells in HCC samples separated by high and low ImmuneRiskScore values.
